# A conserved domain in type III secretion links the cytoplasmic domain of InvA to elements of the basal body

**DOI:** 10.1107/S0907444910010796

**Published:** 2010-05-15

**Authors:** Mirjana Lilic, Cindy M. Quezada, C. Erec Stebbins

**Affiliations:** a Laboratory of Structural Microbiology, The Rockefeller University, New York, NY 10065, USA

**Keywords:** *Salmonella*, type III secretion, bacterial pathogenesis, protein translocation

## Abstract

Protein type III secretion systems (T3SSs) are organic nanosyringes that achieve an energy-dependent translocation of bacterial proteins through the two membranes of Gram-negative organisms. Examples include the pathogenic systems of animals, plants and symbiotic bacteria that inject factors into eukaryotic cells, and the flagellar export system that secretes flagellin. T3SSs possess a core of several membrane-associated proteins that are conserved across all known bacterial species that use this system. The *Salmonella* protein InvA is one of the most highly conserved proteins of this core of critical T3SS components. The crystal structure of a C-­terminal domain of InvA reveals an unexpected homology to domains that have been repeatedly found as building blocks of other elements of the T3SS apparatus. This suggests the surprising hypothesis that evolution has produced a significant component of the apparatus structure through a series of gene-duplication and gene-rearrangement events.

## Abstract 2

The cytoplasmic domain of *Salmonella* InvA shares homology to a recurring scaffold in the membrane-spanning components of the type II and type III secretion systems.

## 1. Introduction

A number of Gram-negative bacterial pathogens, including those causing disease in animals as well as in plants, utilize a highly specialized nanomachine termed the type III secretion system (T3SS) to achieve a remarkable translocation of bacterial proteins across three membranes and directly into the cytoplasm of the host organism (Galan & Wolf-Watz, 2006 ▶; Cornelis, 2006 ▶). These virulence proteins, often called ‘effectors’, hijack eukaryotic biochemical processes in sophisticated ways for the benefit of the pathogen (Cunnac *et al.*, 2009 ▶; Parsot, 2009 ▶; McGhie *et al.*, 2009 ▶; Poueymiro & Genin, 2009 ▶; Galan, 2009 ▶). The secretion machinery itself appears to be highly conserved between different bacteria (Galan & Wolf-Watz, 2006 ▶; Cornelis, 2006 ▶; Marlovits & Stebbins, 2010 ▶).

The engine of this complicated ‘molecular syringe’ consists of a set of proteins in the inner membrane of these Gram-negative organisms and extends into the cytoplasm, including an ATPase and several key transmembrane proteins (Moraes *et al.*, 2008 ▶; Marlovits & Stebbins, 2010 ▶). InvA is a member of a set of several inner membrane proteins that form this core of the T3SS. Highly conserved across pathogenic bacteria, as well as with a conserved homolog in the flagellar system (FlhA), InvA is critical to the functioning of the T3SS (Galan *et al.*, 1992 ▶; Ginocchio & Galan, 1995 ▶). However, the role of InvA, why it is important and how it functions in the T3SS remains completely unknown. Apart from its sequence similarity to analogous components of other T3SSs, InvA shows no primary sequence similarity to any proteins of known function.

To begin to address some of these outstanding questions, we determined the crystal structure of a C-terminal fragment of InvA to 1.9 Å resolution. This structure revealed the unexpected result that a set of structural domains that repeat in the proteins forming the basal body are also present in InvA, suggesting that large portions of the T3SS have been con­structed from an evolutionarily conserved building block.

## 2. Materials and methods

### 2.1. Cloning, expression and purification of InvA(356–525)

An InvA fragment spanning residues 356–525 was amplified by PCR from *Salmonella* genomic DNA. This domain was ligated into a modified pCDFDuet-1 vector (EMD Chemicals Inc., Gibbstown, New Jersey, USA) containing an affinity tag with 12 consecutive histidines and a 3C protease recognition sequence to remove the tag. The protein was expressed in LB medium containing 50 µg ml^−1^ streptomycin and 1 m*M* iso­propyl β-d-1-thiogalactopyranoside (IPTG) in *Escherichia coli* BL21 (DE3) (Stratagene, La Jolla, California, USA) at 294 K overnight following induction at an OD_600_ of 0.8. Cells were harvested by centrifugation and the pellet was dissolved in a buffer (buffer *A*) consisting of 25 m*M* Tris–HCl pH 8.0, 200 m*M* NaCl, 5 m*M* imidazole pH 8.0, 1 m*M* phenylmethanesulfonyl fluoride (PMSF) and lysed using an Emulsiflex C-5 cell homogenizer (Avestin Inc., Ottawa, Ontario, Canada). The lysate was centrifuged at 16 000 rev min^−1^ and 277 K for 30 min. InvA(356–525) protein was purified on Ni–NTA Sepharose (Qiagen) equilibrated in buffer *A* and was eluted from the column with buffer consisting of 25 m*M* Tris–HCl pH 8.0, 200 m*M* NaCl, 250 m*M* imidazole pH 8.0. Upon cleavage with 3C protease, InvA(356–525) protein was concentrated using a Amicon Ultracell 3K (Millipore) and loaded onto a gel-filtration column (Superdex 200 HighLoad 16/60, GE Healthcare) equilibrated in buffer containing 25 m*M* Tris–HCl, 200 m*M* NaCl, 2 m*M* dithiothreitol (DTT) using ÄKTA FPLC. Selenomethionine-substituted protein was purified as for the unlabeled protein.

### 2.2. Crystallization and structure determination of InvA(356–525)

For crystallization, InvA(356–525) was concentrated to 25 mg ml^−1^ in a buffer consisting of 25 m*M* Tris pH 8.0, 200 m*M* NaCl, 2 m*M* DTT. Crystals were grown by vapor diffusion using hanging drops formed by mixing a 1:1 volume ratio of InvA(356–525) protein solution and equilibration buffer con­sisting of 1.6 *M* ammonium sulfate, 0.1 *M* MES pH 7.0, 8% dioxane at 296 K. For cryoprotection, crystals were transferred directly into buffer consisting of 1.2 *M* ammonium sulfate, 0.1 *M* MES pH 7.0, 7% dioxane, 25% glycerol and flash-cooled to 113 K.

Reproducibility was a significant challenge with the crystals obtained. Many crystals produced data sets that could not be effectively scaled and ‘good’ crystals were very rare amongst the many that were screened. The model was phased and refined against a selenomethionine-substituted crystal which diffracted and processed well. To date, obtaining a well diffracting native data set has proved problematic.

Data were collected on Brookhaven National Synchrotron light source beamline X29 as a single-wavelength anomalous dispersion data set using selenomethionine-substituted protein crystals and were processed using *HKL*-2000 (Otwinowski & Minor, 1997 ▶). To increase the anomalous signal, two additional selenomethionine sites were introduced into InvA(356–525) by site-directed mutagenesis at residues Leu383 and Leu470, and this mutant protein was purified identically to the native. The crystals belonged to space group *I*4, with unit-cell parameters *a* = *b* = 83.7, *c* = 130.4 Å. There were two InvA molecules in the asymmetric unit. Phases were determined using *SHELX* (Sheldrick, 2008 ▶) and *PHENIX* (Adams *et al.*, 2010 ▶) and 90% of the final model was built by *ARP*/*wARP* (Langer *et al.*, 2008 ▶). Cycles of manual building and refinement with *REFMAC*5 (Murshudov *et al.*, 1997 ▶) resulted in a model with an *R* and *R*_free_ of 21.0% and 23.8%, respectively, to 1.85 Å resolution. The data-collection, structure-determination and refinement statistics are summarized in Table 1 ▶.

## 3. Results and discussion

InvA of *S. typhimurium* is 685 amino acids in length and the N-terminal 300 amino acids are comprised of seven transmembrane helices. InvA also possesses a large cytoplasmic domain spanning residues ∼350–685. Both the transmembrane and the cytoplasmic domains have been shown to be critical for T3SS activity (in InvA and in the flagellar homolog FlhA; Ginocchio & Galan, 1995 ▶; McMurry *et al.*, 2004 ▶). The C-terminal domain is not membrane-associated except by its attachment to the N-terminal domain and can be produced separately as a soluble entity.

**Figure 1:**
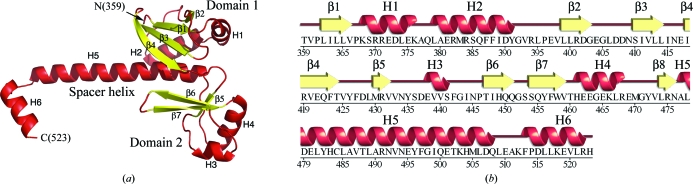
Overall structure of *Salmonella* InvA(359–523). (*a*) Overall fold of the InvA(359–523) monomer. Helices are shown in red and strands are shown in yellow. The two domains are labeled, as are the spacer helix, the individual elements of secondary structure and the termini of the construct. (*b*) Sequence and secondary-structural diagram of the InvA(359–523) monomer.

Constructs of the cytoplasmic domain of InvA that spanned the entire C-­terminal sequence following the pre­dicted transmembrane regions proved to be highly soluble and stable (data not shown), but were recalcitrant to crystallization. A series of limited proteolytic digestions coupled with Edman sequencing and mass-spectrometric analysis identified several possible sub­domains that were more amenable to crystallization. The construct 356–525 produced crystals that diffracted well and allowed the high-resolution structural determination of roughly half of the InvA cytoplasmic domain (Table 1 ▶ and Figs. 1 ▶, 2 ▶ and 3 ▶).

### 3.1. Overall structure of InvA(356–525)

The structure of InvA(356–525) reveals three subdomains: two globular folds at the N-terminal end of the construct followed by a long helix (spacer helix) that would presumably lead to the far C-terminal subdomain(s) of the cytoplasmic region (Figs. 1 ▶ *a* and 1 ▶ *b*). The far C-terminal helix may in fact represent a portion (the beginning) of the subdomain fold that is missing from the crystallized construct. Barring a conformation change, this arrangement suggests that there are likely to be two globular regions in the InvA cytoplasmic portion that are spatially separated through the spacer helix. It is also possible, however, that the far C-terminal region could travel back and pack against the globular portion of the construct that we have crystallized.

This domain of InvA is present as a dimer in the asymmetric unit of the crystals, related by a twofold axis of symmetry. The biological significance of this dimer is uncertain, as the protein migrates as a monomeric species on size-exclusion chromatography and the buried surface area of this interaction is less than 500 Å^2^. The two copies of the protein are very similar, with a root-mean-square deviation in C^α^ positions of 0.8 Å. Two regions of the protein contribute most to this variation: the C-terminal helix of the truncated construct and an extended region of 30 amino acids spanning residues 370–400. In this latter extended region, covering two helices and a loop, there are overall small translational shifts in the helices in the alignment and in once place, involving residues 390–399, the long loop between H2 and β2 adopts very different conformations, both of which are well ordered in the electron-density maps.

### 3.2. Presence of T3SS conserved folds

**Figure 2:**
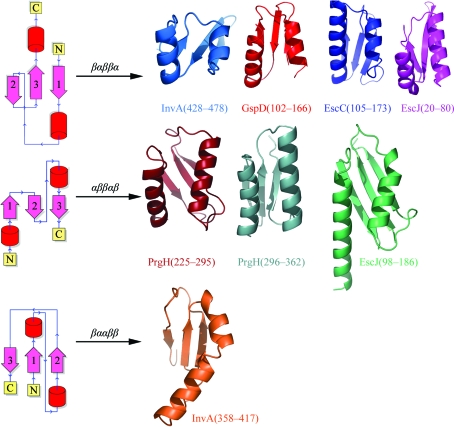
Conserved fold in multiple elements of the T3SS. Shown along with the two InvA domains are the folds of GspD, EscJ, EscC and PrgH (PDB codes 3ezj, 1yj7, 3gr5 and 3gr0, respectively; Korotkov *et al.*, 2009 ▶; Yip *et al.*, 2005 ▶; Spreter *et al.*, 2009 ▶) divided into three classes based on protein fold. On the left is a topology diagram for each class to illustrate the connectivity differences that are present in these similar-appearing folds.

When the InvA structure is compared with structures deposited in the Protein Data Bank (PDB), there is a surprising finding. The subdomain spanning residues 428–478 possesses homology to the inner membrane ring-forming protein EscJ (PrgK in *Salmonella*; Fig. 2 ▶). This domain also appears in the outer membrane secretin ring, EscC, in the *E. coli* T3SS and GspD of the type II secretion system (Spreter *et al.*, 2009 ▶). The recurrence of this fold in these ring-forming proteins has led to the hypothesis that the domain itself is a ‘ring-forming’ motif (Spreter *et al.*, 2009 ▶). However, InvA is not known (or hypothesized) to form a ring and the packing of these two domains in the InvA crystals differs markedly from that of the EscJ tetramer that was used to model the ring (Yip *et al.*, 2005 ▶).

As has been noted (Marlovits & Stebbins, 2010 ▶), the recurring three-dimensional folds in all of the ring-forming proteins of the T3SS appear to be superficially similar: there is a three-stranded β-sheet core with two antiparallel and interacting helices on one face of the sheet that run parallel to the strands. Visually, the overall folds are superimposable. The folds are distinct, however, in their topology (Fig. 2 ▶), with one domain having a βαββα fold (strand–helix–strand–strand–helix) and the other having an αββαβ fold (helix–strand–strand–helix–strand). The second InvA subdomain falls into the former class.

Interestingly, the first InvA subdomain (residues 358–417) also has a core with two α-helices and a three-stranded β-­sheet, two strands of which are parallel. However, its topology is distinct from the two classes mentioned above as the strands possess different connectivity and the helices are arranged very differently in three-dimensional space. Searches of the PDB (Holm *et al.*, 2008 ▶) return homology to a fold found in diverse proteins of seemingly unrelated function, such as the ‘small domain’ of bacterial RNase E and a portion of MIF (macrophage migration inhibitory factor; PDB entries 2vmk and 2wkb; Koslover *et al.*, 2008 ▶; Dobson *et al.*, 2009 ▶).

### 3.3. Tetrameric InvA and comparisons to ring-forming proteins

**Figure 3:**
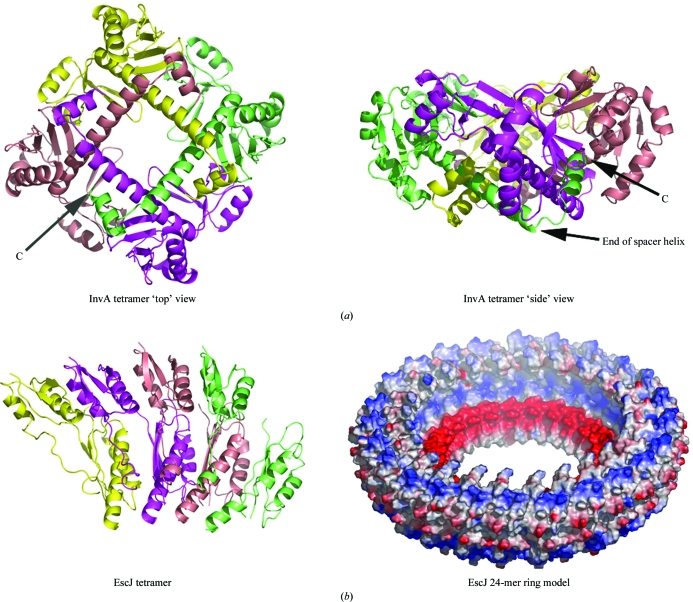
Oligomeric assemblies of InvA and EscJ. (*a*) The crystallographic tetrameric assembly of the crystallized InvA construct is shown in two views side by side, related by a 90° rotation about a vertical axis. Each of the chains is given a separate color and the COOH-terminus of the green polypeptide is shown; the spacer helix is marked on the right image. (*b*) The EscJ tetramer present in the asymmetric unit of the crystals, alongside a surface representation (colored by electrostatic potential) of the 24-mer ring model of EscJ proposed to form a portion of the inner membrane ring of the basal body of the type III secretion system.

InvA(356–525) is present as a noncrystallographic dimer in the crystals and forms a tetramer in crystal packing (Fig. 3 ▶), despite the fact that this construct elutes from gel-filtration chromatography as a monomer (data not shown). The tetramer is highly interdigitated, burying a total of nearly 10 000 Å^2^ of surface area (Fig. 3 ▶). Unlike the EscJ tetramer, this tetramer is rotationally symmetric and there is no clear manner in which it could be modeled as a ring along the lines of the inner membrane ring of the basal body. Despite its extensive contacts, the biological significance of the crystallo­graphic tetramer is uncertain. Whether the entire C-terminal domain can adopt a similar tetrameric arrangement is unclear and the packing could be a result of the truncation of the domain and/or crystal packing. There is little support for the tetramer outside of the crystals, as this construct, as well as the entire C-terminal domain, run as monomers on gel-filtration chromatography.

## 4. Conclusions

T3SSs of Gram-negative bacteria are the critical virulence devices of a large number of medically and agriculturally relevant pathogens. Structural insight into this virulence system will fill an important gap in the knowledge of infectious agents, as well as providing blueprints for the targeted disruption of this system, potentially by therapeutic com­pounds.

The structure of InvA reported here reveals an unexpected homology to domains that have recently been shown to be present in the channel-forming proteins of the pathogenic T3SS. The presence of this fold in all of these elements of the secretion machinery, from membrane-spanning channels to soluble cytoplasmic components, indicates that the protein T3SS has evolved in part from a set of common ‘bricks’: proteins encoded by genes that are likely to be the result of duplication and divergence. That the megadalton-sized nanosyringe could be so constructed is a fascinating surprise, illustrating the economy of biological evolution.

## Supplementary Material

PDB reference: cytoplasmic domain of InvA, 3lw9

## Figures and Tables

**Table 1:** Data-collection and refinement statistics for SeMet InvA(356–525) Values in parentheses are for the high-resolution shell (1.92–1.85 Å).

Data collection	
Space group	*I*4
Unit-cell parameters (Å, °)	*a* = *b* = 83.7, *c* = 130.4, α = β = γ = 90.0
Resolution (Å)	19.29–1.85
No. of reflections	1313988
No. of unique reflections	75386
*R*_merge_†	6.5 (77.7)
*I*/σ(*I*)	29.3 (2.1)
Completeness (%)	99.8 (100.0)
Redundancy	7.6 (7.6)
Refinement	
Resolution (Å)	19.29–1.85
No. of reflections	36968
*R*_work_/*R*_free_‡ (%)	21.0/23.8
No. of atoms	
All atoms	2916
Protein	2745
Water	171
*B* factors (Å^2^)	
All atoms	29.4
Protein	29.1
Water	33.7
R.m.s. deviations from ideal values	
Bond lengths (Å)	0.016
Bond angles (°)	1.519

† *R*_merge_ = 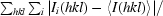 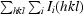 for the intensity (*I*) of *i* observations of reflection *hkl*.

‡ *R* = 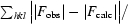 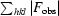, where *F*_calc_ is the model structure factor and 5% data were omitted for calculation of *R*_free_.
